# Sequences of synaptogenesis in the human fetal and neonatal brain by synaptophysin immunocytochemistry

**DOI:** 10.3389/fncel.2023.1105183

**Published:** 2023-02-03

**Authors:** Harvey B. Sarnat

**Affiliations:** Department of Paediatrics (Neurology), Pathology and Laboratory Medicine (Neuropathology) and Clinical Neurosciences, University of Calgary Cumming School of Medicine and Alberta Children’s Hospital Research Institute (Owerko Centre), Calgary, AB, Canada

**Keywords:** synaptogenesis, fetus, neonate, synaptophysin, brain development

## Abstract

Synaptogenesis is the final phase of axonal pathfinding. Its sequences of spatial and temporal development in the immature nervous system are precisely timed and consistent. Synaptophysin, a principal structural glycoprotein of synaptic vesicle membranes regardless of the chemical transmitter substance within, is a reliable means of demonstrating sequences of synaptogenesis in human fetal brain tissue at autopsy and is resistant to postmortem autolysis. Furthermore, synaptophysin molecules are demonstrated during axoplasmic flow before being assembled into membranes in immature axons and also mature axons of neurons with a high metabolic rate. In brain malformations these sequences often are altered both in distribution of synapses and in timing, often delayed but sometimes precocious, with postnatal clinical manifestations such as epilepsy and cognitive development.

## Introduction

Synaptogenesis is fundamental to nervous system circuitry and networks that are the basis of functional expression. Though the concept had been expressed decades earlier (Ariëns Kappers, 1917), chemotropic guidance of axons to their target neurons for synapse formation was better understood in the 1980s (Brown et al., 2001; Rao and Jacobson, 2005; Sarnat, 2023). The final phase of axonal pathfinding as the axonal tip or growth cone approaches its target is terminal ramification of the growing axon and synapse formation on target neurons.

Synaptophysin is a structural glycoprotein, a major constituent of synaptic vesicular membranes, regardless of the identity of neurotransmitter within the vesicles in axonal terminals. Other structural glycoproteins of these vesicular membranes include synaptobrevin and synaptotagmin. The latter is particularly important for rapid resupply of synaptic vesicles by micromolar presynaptic calcium signals (Weingarten et al., 2022). Synaptophysin was first discovered and then applied by immunocytochemistry to sections of mature human brain in the late 1980s (Jahn et al., 1985). Immunoreactivity of synaptophysin later enabled the demonstration of sequences of synaptogenesis in the cerebral cortex and hippocampus, temporally coinciding with and explaining EEG maturation in preterm neonates (Sarnat and Born, 1999; Sarnat et al., 2010). Cortical lamina-specific synaptic connections were demonstrated in the late 1990s (Sanes and Yamagata, 1999) and other layer-specific markers also were identified shortly thereafter (Hevner, 2007). After axoplasmic transport from its site of synthesis in the perinuclear cytoplasm, within the terminal axon synaptophysin combines with other structural proteins such as synaptobrevin, synaptotagmin, and SNAP25 to form the membranes of synaptic vesicles by processes both involving and also independent of exocytosis (Jahn and Südhof, 1994; Edelman et al., 1995; Reisinger et al., 2004). Synaptophysin also relates to non-structural soluble proteins of the synaptic vesicle such as synapsin-1 and -2 (Thiel, 1993). Synaptophysin immunoreactivity is resistant to postmortem autolysis for up to 5 days, hence is useful in autopsy studies of standard formalin-fixed fetal and neonatal brain sections cut from paraffin-embedded blocks (Sarnat et al., 2010).

Timing is a key determinant not only of normal brain development but aIso cerebral dysgeneses (Sarnat and Flores-Sarnat, 2014). In malformations of cortical development, synaptophysin enables the demonstration of precocious and abnormally distributed synaptogenesis, such as in lobar/semilobar holoprosencephaly (Sarnat and Flores-Sarnat, 2013) and delayed synaptogenesis in many other genetic and metabolic encephalopathies beginning prenatally. Pathological synapse formation can exhibit shifts in synaptic ratios (proportions of excitatory and inhibitory influences) helping to explain why some cerebral malformations are more epileptogenic than other (Sarnat and Flores-Sarnat, 2021).

Synaptophysin shows that synapse formation normally precedes mature morphological form in some structures, such as the dentate nucleus of the cerebellum and inferior olivary nucleus, thus facilitating better interpretation of function in malformations (Sarnat et al., 2013a). Abnormal or excessive synaptic pruning and remodeling also were demonstrated by Golgi impregnations early in the 20^th^ century (Ramón y Cajal, 1995) and by electron microscopy during the mid-20th century, described in 1956 by Palay (1956) and a little later by Collonier (1968), and Molliver et al. (1973). The ultrastructural demonstration of the synaptic cleft and pre- and postsynaptic membrane specializations also definitively confirmed the neuronal hypothesis of Ramón y Cajal which was intensely debated in the late 19th and early 20th centuries against the prevailing belief that the central nervous system was a syncytium of continuous processes, analogous to the root systems of many mushrooms, dandelions and shrubs.

Whereas synaptophysin immunoreactivity can be studied by itself, it is best understood in the context of simultaneous other immunocytochemical reactivities that identify neuronal maturation (Lyck et al., 2008; Sarnat, 2013, 2015). Photomicrographic illustrations in color of the various topics discussed below are found in each of the cited references.

### Sequences of synaptogenesis in human fetal brain

The following below description summarizes studies in our laboratory of the sequences of synaptogenesis in various structures of the human fetal and neonatal brain, as demonstrated at autopsy in paraffin-embedded tissue sections by anti-synaptophysin immunocytochemical antibody (Novocastra Laboratories, Newcastle upon Tyne, UK; distributed through Vison Biosystems, Norwell, MA, USA. NCL-SYNAP-299; 1:100 dilution with thermal intensification). In our studies of more than 200 normal human fetal and neonatal brains at various gestational ages from 6 to 42 weeks, we found reliable consistency between brains of similar age in distribution and timing of appearance of synaptophysin reactivity. Other systematic surveys of temporal and spatial sequences of synaptogenesis are sparse, though many neuropathologists in other centers in Canada and Europe have anecdotally confirmed to us their similar findings. Ongoing studies in our laboratory include the spinal cord, retina and detailed nuclei of the brainstem and thalamus.

#### Hippocampus

**FIGURE 1:**
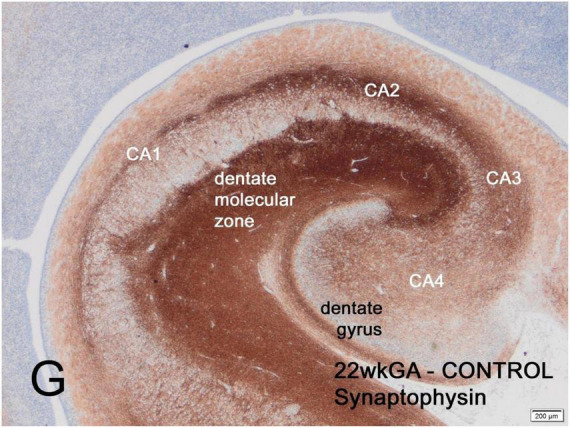
Transverse section of normal hippocampus of a 22-week human fetus showing intense synaptophysin reactivity in the molecular zone of the dentate gyrus and in the CA2 sector of Ammon’s horn. Weaker reactivity is observed in other CA sectors. At term, synaptophysin reactivity is seen uniformly throughout the hippocampus (not illustrated). Reproduced from reference (Sarnat, 2019).

The first part of the human fetal hippocampus to express synaptophysin immunoreactivity is the molecular zone of the dentate gyrus, as early as 12 weeks gestation, followed by the CA2 sector of Ammon’s horn at 14 weeks, CA2 at 14 weeks, CA3 and CA4 at 15-16 weeks and finally CA1 at 19 weeks (Figure 1). Reactivity remained incomplete until 26 weeks but by term was similar to the adult hippocampus (Sarnat et al., 2010).

#### Cerebral cortex

**FIGURE 2:**
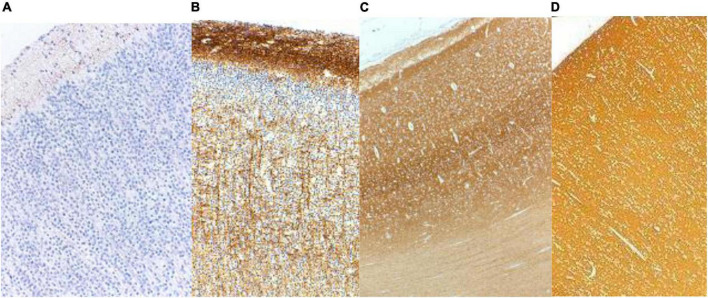
Synaptophysin reactivity in the human cerebral cortex at **(A)** 20 weeks; **(B)** 26 weeks; **(C)** 33 weeks; and **(D)** 40 weeks gestational age. At mid-gestation (20 weeks) only minimal reactivity is seen in transverse axons of Cajal-Retzius neurons of the molecular zone. At 26 weeks the molecular zone and deep cortical layers are reactive, superficial layer 2 is still non-reactive, and coarse radial thalamo-cortical axons also are evident. At 33 weeks the deeper layers of cortex are more intensely reactive than are the superficial layers. At term the cortex is uniformly reactive throughout. Reproduced from reference (Sarnat et al., 2010).

In the frontal lobe, synaptophysin was detected in a laminar pattern above and below the neocortical plate at 12 weeks and around Cajal-Retzius neurons of the molecular zone at 14 weeks gestational age. Pyramidal neurons of the future deep layers of cortex were surrounded by synaptophysin reactivity at 16 weeks and at the surface of neuronal somata of layers 2 and 4 at 22 weeks. Immunoreactivity remained more intense in the molecular zone and in deep cortical layers until about 36 weeks and in late gestation and at term was uniform throughout the cortex (Figures 2A–D; Sarnat et al., 2010). Other lobes of the neocortex were similar though somewhat more delayed in the parietal and temporal lobes. Synaptophysin reactivity is seen in the axoplasm of thalamocortical projections from 23 to 34 weeks gestation (Figure 2C).

#### Olfactory bulb and tract

From 16 weeks gestation synaptophysin immunoreactivity is demonstrated at the somata of mitral and tufted neurons and also many but not all olfactory bulb glomeruli. Glomeruli are uniformly strongly reactive by 20 weeks. The granular layer of the core of the olfactory bulb exhibits concentric alternating layers of neurons and sheets of dendro-dendritic synapses which are synaptophysin immunoreactive in the most peripheral layers from 16 weeks but the deepest third of the multiple layers are still not reactive at term and acquire synaptophysin reactivity in the first few postnatal weeks (Sarnat and Yu, 2016). NeuN, a later neuronal marker of maturation, also is not yet reactive in the nuclei of granular neurons in the deep layers where synaptophysin reactivity has not yet appeared. The granular layer extends into the olfactory tract and timing of reactivity is similar to the superficial granular cell layers of the olfactory bulb. Nodules of the anterior olfactory nucleus also is seen within the olfactory tract and is strongly reactive at mid-gestation. Immunoreactivity with antibodies to SNAP-25, another structural protein of synaptic vesicles, in the full-term fetus were reported to be similar to adult reactivity (Kharlamova et al., 2010), by contrast with the immaturity of synaptophysin expression in deep granular cell layers of the term neonate that we demonstrated.

#### Basal ganglia (corpus striatum; globus pallidus; substantia nigra)

The corpus striatum (caudate nucleus and putamen) shows a unique patchy pattern of synaptophysin reactivity, neither laminar nor uniform, not seen in the normal neocortex but found also in the nuclei of the basis pontis (see cerebellar system below). This patchy synaptophysin pattern of the corpus striatum is first detected at 13 weeks gestation, is prominent before midgestation and is still subtle even at term (Sarnat et al., 2013b). This synaptic patchiness in the fetal corpus striatum appears to correspond to the “striosomes of Graybiel” that define adjacent neurotransmitter-rich and neurotransmitter-poor zones (Graybiel et al., 1979, 1981; Graybiel, 1982). Clinical correlation is proposed with dystonic postures and athetoid movements observed in normal preterm neonates of 26 to 32 weeks (Sarnat et al., 2013b). The globus pallidus already shows uniform synaptophysin reactivity from 13 weeks gestation, more intense with advancing gestational age (Sarnat et al., 2013b). Synaptophysin reactivity is demonstrated in the substantia nigra of the midbrain from 9 weeks gestation, intense by 14 weeks.

#### Thalamus

Ascending thalamocortical axons were detected with axoplasmic synaptophysin reactivity as early as 12 weeks but most intense and best demonstrated at 26 weeks gestation (Sarnat et al., 2010). Thalamic neurons and neuropil were strongly reactive by 15 weeks gestation, but individual thalamic nuclei were not distinguished by synaptophysin. Neuronal markers neuronal nuclear antigen (NeuN), calretinin, neuron-specific enolase (NSE) and neurofilament protein (NFP) also are strongly reactive. In the second trimester the thalamus and globus pallidus also are the two regions of the brain strongly expressing keratan sulfate immunoreactivity, an extracellular axonal guidance molecule secreted by astrocytes (Sarnat, 2019).

#### Cerebellar system

The cerebellar cortex is best considered not in isolation but as part of circuits that comprise complex cerebellar systems. One loop, limited to brainstem structures, is the “Guillain-Mollaret triangle” or dentato-rubro-olivary-cerebellar circuit. Another is the ascending dentato-ventrolateral thalamo-cortical-pontine-cerebellar circuit. Prominent dorsal and ventral ascending spinocerebellar tracts are present, but a reciprocal direct cerebello-spinal pathway is lacking, though the cerebellum can influence the spinal cord via small vestibulospinal, reticulospinal, olivospinal and tectospinal tracts. The principal influence of the cerebellum on the spinal cord for muscle tone and coordination is via the corticospinal tract.

The dentate and other smaller deep cerebellar nuclei (fastigium; interpositum; globosus), as well as the inferior olivary nuclei, initially are rather amorphous gray matter masses without much form; only with maturation do they convolute to form the “crenated” characteristic form of the mature brain. Synaptophysin reactivity appears in these nuclei before crenation occurs, highlighting a fundamental principle of developmental neuroanatomy that *neuronal maturation and synaptogenesis can occur before the mature anatomical form of the nuclei develops*. Crenation of these nuclei begins just before mid-gestation and is complete by 33 weeks, similar to the adult morphology.

**FIGURE 3:**
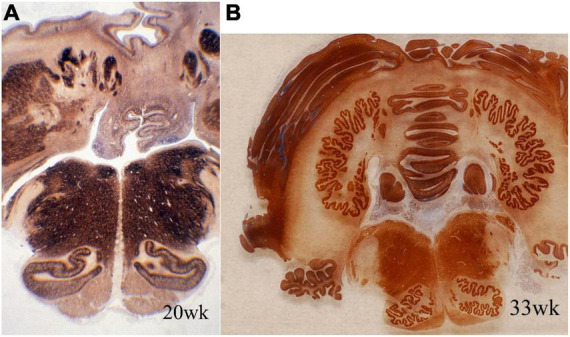
Transverse sections of cerebellum and medulla oblongata of normal human fetuses of **(A)** 20 weeks and **(B)** 33 weeks gestation, showing synaptophysin immunoreactivity. The dentate nucleus and smaller deep cerebellar nuclei are amorphous in form at mid-gestation and exhibit the normal crenated adult form by 33 weeks gestation. Synaptogenesis already is evident before the mature morphology is achieved. The inferior olivary nuclei are a primitive C-shape at 20 weeks and are not crenated, but have the mature convoluted shape by 33 weeks. Synaptophysin is seen in the periphery of these nuclei but only weakly in the center at mid-gestation. Reproduced from reference (Sarnat et al., 2013a).

Accessory olivary and deep cerebellar nuclei are intensely reactive for synaptophysin earlier than the principal olivary and dentate nuclei (Figure 3). The dorsal blades of both are formed earlier than the ventral, with reactivity initially peripheral and only later within the deep cores of the nuclei. Initiation of synaptophysin reactivity is at 13 weeks in the inferior olivary and at 16 weeks in the dentate nuclei (Sarnat et al., 2013a).

The red nucleus matures in synaptophysin reactivity later than the bulbar nuclei. Before 18 weeks, the red nucleus contrasts with the intensely reactive surrounding tegmental gray matter because of its lack of reactivity. Later in gestation it also exhibits high contrast. The transition is at about 18 weeks. Synaptophysin reactivity in the red nucleus is initiated in the periphery, with scattered nests of reactive small neurons also in its more central and inferior region where large neurons (magnocellular) predominate. The lesser reactivity seen in the center than the periphery persists until term but part of this appearance is because neurons normally are less densely clustered in the center than the periphery of the red nucleus (Sarnat et al., 2013a). Histologically, the pontine nuclei neurons appear uniform without a hint of the patchy synaptophysin pattern.

Pontine nuclei of the basis pontis receive corticopontine axons from neocortex and project their axons to the contralateral cerebellar cortex. As noted above, as with the corpus striatum, these nuclei have patchy synaptophysin reactivity not explained by intervening axonal projections of the corticospinal and corticopontine pathways. Its synaptic vesicle reactivity appears at 20 weeks and is uniform by 34 weeks gestation (Sarnat et al., 2013c).

In the cerebellar cortex, the vermis matures sooner than the cerebellar hemispheres and the paravermal portions earlier than the lateral folia. The earliest synapses occur around the somata of Purkinje neurons and later in the internal granular layer, but synaptic glomeruli are not well formed until after 26 weeks (Sarnat et al., 2013c). In the external granular layer of the cerebellar cortex, synaptophysin reactivity can be demonstrated in the deep layer of premigratory granular neurons that already have extended their axons into the molecular zone for synapse formation with Purkinje cell dendrites, but not in the outer layer of the external granular zone where mitotic activity is still present. The molecular zone also exhibits synaptophysin reactivity.

#### Area postrema

**FIGURE 4:**
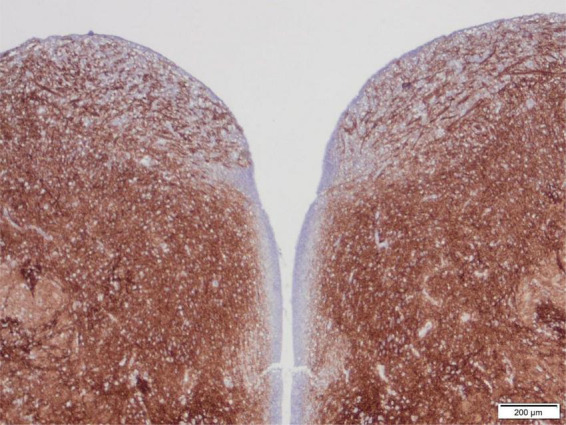
Transverse section of the normal tegmentum of the medulla oblongata at the level of the caudal end of the 4th ventricle, of a 15-week human fetus, showing synaptophysin immunoreactivity. The dorsal regions with coarse axons and small neurons just beneath the ventricle on either side are the area postrema. The tegmentum otherwise showed intense reactivity in all gray matter regions but not in longitudinal white matter tracts such as the tractus solitarius at the lateral edge of the section, with strong reactivity surrounding this fascicle as the nucleus of the tractus solitarius. Reproduced from reference (Sarnat et al., 2019).

The area postrema is a small but important nucleus in the dorsomedial medulla oblongata near to the caudal end of the floor of the 4th ventricle (Figure 4), with rather widespread connections. It shows strong immunoreactivity for synaptophysin from 14 to 15 weeks gestation, but never as intense as many nuclei of the tegmentum of the brainstem, in part because the area postrema is a highly vascular structure without a blood-brain barrier (Sarnat et al., 2019).

#### Nucleus of the tractus solitarius

The tractus solitarius is a paired small descending pathway in the tegmentum of the medulla oblongata, surrounded by neurons of its nucleus (Figure 4). Together with the pre-Bötzinger nucleus located more ventrally, it is the principal respiratory center of the brainstem. Synaptogenesis is first demonstrated by synaptophysin antibodies in this nucleus at 12 weeks gestation and matures by 15 weeks; myelination, by contrast, is not initiated in the tracts solitarius until 33 weeks (Sarnat and Flores-Sarnat, 2016).

#### Synaptic plexi in U-fiber layer

**FIGURE 5:**
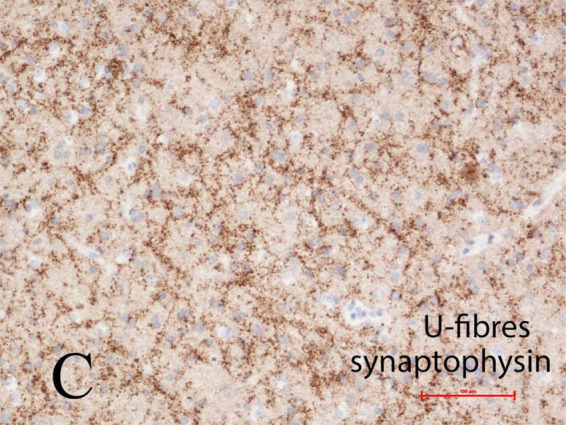
Complex synaptic plexi are formed between heterotopic neurons in the U-fiber layer beneath the cortex in normal brain but are greatly increased beneath focal cortical dysplasias (FCD) types I or II, as here demonstrated with synaptophysin immunoreactivity at the base of a gyrus in a 4-year-old girl with FCD type IIa. These plexi also integrate with overlying cortex and thus may contribute to epileptic networks. Reproduced from reference (Sarnat et al., 2018).

The U-fiber layer is a rim of white matter just beneath layer 6 of the neocortex and follows the contours of the gyri. U-fibers consist of short association axons of neurons in layer 6 that locally interconnect different parts of the same gyrus and also immediately adjacent gyri, but are not long descending projections or commissural fibers, thus differ significantly from the deep white matter of the cerebral hemisphere. The U-fiber layer develops in fetal life when gyration and sulcation of the cortex begin, shortly after mid-gestation. Within the U-fibers of normal mature brains are a few heterotopic neurons with axonal connections between them. These axons can be demonstrated by synaptophysin immunoreactivity. Beneath focal cortical dysplasias, types I and II, the number of heterotopic neurons is greatly increased and complex synaptic plexi form between them and also extending into the overlying cortex to integrate with epileptic networks (Sarnat et al., 2018). These plexi are well demonstrated by synaptophysin in tissue sections at any age including older children and adults, suggesting that these neurons have a high metabolic and discharge rate (Figure 5).

## Conclusion

Synaptophysin immunocytochemistry is a reliable and valuable method of demonstrating sequences of normal and abnormal synaptogenesis in paraffin sections of the various structures of human fetal and neonatal brain. It adds a dimension of understanding to neuropathological examinations by light microscopy of immature brain tissue not duplicated by histological stains, particularly useful in studying cerebral malformations. It is useful both for research and in diagnostic neuropathology.

## Author contributions

The author confirms being the sole contributor of this work and has approved it for publication.
